# Can Maternal Exposure to Air Pollution Affect Post-Natal Liver Development?

**DOI:** 10.3390/toxics11010061

**Published:** 2023-01-09

**Authors:** Yong Song, Ling Chen, Ellen Bennett, Amanda J. Wheeler, Katherine Southam, Seiha Yen, Fay Johnston, Graeme R. Zosky

**Affiliations:** 1 Menzies Institute for Medical Research, College of Health and Medicine, University of Tasmania, Hobart, TAS 7001, Australia; 2 School of Biomedical Sciences and Pharmacy, University of Newcastle, Callaghan, NSW 2308, Australia; 3 Hunter Medical Research Institute, New Lambton Heights, NSW 2305, Australia; 4 Tasmanian School of Medicine, College of Health and Medicine, University of Tasmania, Hobart, TAS 7000, Australia; 5 Commonwealth Scientific and Industrial Research Organisation, Aspendale, VIC 3195, Australia

**Keywords:** liver development, maternal exposure, particulate matter, genotoxicity

## Abstract

Emerging evidence suggests that inhalation of particulate matter (PM) can have direct adverse effects on liver function. Early life is a time of particular vulnerability to the effects of air pollution. On that basis, we tested whether in utero exposure to residential PM has an impact on the developing liver. Pregnant mice (C57BL/6J) were intranasally administered 100 µg of PM sampled from residential roof spaces (~5 mg/kg) on gestational days 13.5, 15.5, and 17.5. The pups were euthanized at two weeks of age, and liver tissue was collected to analyse hepatic metabolism (glycogen storage and lipid level), cellular responses (oxidative stress, inflammation, and fibrosis), and genotoxicity using a range of biochemical assays, histological staining, ELISA, and qPCR. We did not observe pronounced effects of environmentally sampled PM on the developing liver when examining hepatic metabolism and cellular response. However, we did find evidence of liver genomic DNA damage in response to in utero exposure to PM. This effect varied depending on the PM sample. These data suggest that in utero exposure to real-world PM during mid-late pregnancy has limited impacts on post-natal liver development.

## 1. Introduction

Inhalation of air pollution has detrimental impacts on the respiratory, cardiovascular, and central nervous systems [1,2]. In line with these direct effects, gestational exposure to particulate matter (PM), a key component of air pollution, can have an impact on post-natal development through reduced post-natal somatic growth, impaired lung and immune development, and neuropathological changes [3,4,5]. The mechanisms underlying these post-natal effects are not fully understood but are likely to be related to direct tissue damage by particle translocation through the placenta or indirectly by upregulation of signalling molecules released as part of the maternal inflammatory response [6].

In addition to the well-documented adverse effects on the lung, heart, and brain, epidemiological evidence is emerging to suggest that air pollution exposure can impact other organs such as the liver by increasing the risk of fatty liver disease [7] and liver cancer [8,9]. These observations are supported by work in animal models showing liver toxicity and inflammation following exposure to air pollutants [10]. For example, exposure to airborne fine PM for 10 weeks induced hepatic steatosis and inflammation and promoted hepatic fibrogenesis in a murine model [11,12]. Similarly, PM exposure for 24 h in rats led to oxidative stress, inflammation, genotoxicity, and DNA repair [13]. However, the potential impact of early-life exposure to PM on the developing liver is largely unknown. Although one study showed that in utero exposure to pure carbon black nanoparticles induced post-natal DNA damage in the liver of exposed offspring [6].

Given that the developing foetus is particularly vulnerable to environmental stressors and that the effects of exposure in early life can have lifelong impacts, it is imperative to understand how indoor PM, particularly given pregnant women may spend >80% of the day in this environment [14], impacts on fetal development. In this regard, we have previously shown that maternal exposure to indoor PM impairs post-natal lung, brain, and immune development [4,5]. Using our well-established mouse model of in utero PM exposure, we aimed to determine whether maternal exposure to real-world indoor PM affects post-natal liver development. We evaluated the hepatotoxic effects of PM exposure on the developing liver by examining hepatic metabolism, the cellular response (oxidative stress, inflammation, and fibrosis), and genotoxicity, all of which have been strongly associated with liver injury caused by direct exposure to air pollutants [10].

## 2. Materials and Methods

### 2.1. Particles and Animal Exposure

Detailed information regarding the PM collection and characterization, as well as the animal exposure protocol, has been reported elsewhere [4,5]. Briefly, particle samples were collected from the residential roof areas of three houses (samples 1–3). Twenty-three common elements (Li, Be, V, Cr, Mn, Co, Ni, Cu, Zn, As, Se, Mo, Cd, Sb, Ba, Pb, Na, Mg, Ca, K, Fe, Al, and S) were analysed in particle samples using inductively coupled plasma mass spectrometry and inductively coupled plasma optical emission spectrometry. The dominant elements in the PM varied between samples (particularly between sample 1 and samples 2–3). For example, Ca (1.92 × 10^4^ mg/kg for sample 1; 3.31 × 10^4^ mg/kg for sample 2; and 3.93 × 10^4^ mg/kg for sample 3), Al (2.00 × 10^4^ mg/kg for sample 1; 1.68 × 10^4^ mg/kg for sample 2; and 1.52 × 10^4^ mg/kg for sample 3), and Zn (3.33 × 10^4^ mg/kg for sample 1; 308 mg/kg for sample 2; and 982 mg/kg for sample 3). The polycyclic aromatic hydrocarbon (PAH) content in the PM was also analysed using gas chromatography mass spectrometry. PAH levels were relatively consistent between samples.

Pregnant mice (C57BL/6J) were intranasally administered 100 µg particles (~5 mg/kg) in saline (50 µL), or saline (50 µL) alone as controls, after light methoxyflurane anaesthesia through mid-to-late gestation (gestational days 13.5, 15.5, and 17.5), which is regarded as a window of sensitivity to the effects of air pollution on the development of multiple systems [4,5,15,16,17,18]. This dose and delivery route have been validated in a previous study by our group [5]. The pups were sacrificed at two weeks of age by sodium pentobarbitone injection, giving us four groups of mice: saline; PM sample 1; PM sample 2; and PM sample 3. All experiments involving mice received approval from the University of Tasmania Animal Ethics Committee (Ethics No. A0015505) and abided by the National Health and Medical Research Council (Australia) code of conduct. Liver samples were dissected from the pup, snap-frozen in liquid nitrogen, and then stored at −80 °C for later analysis.

### 2.2. Protein Assay

Total protein was quantified in tissue lysates using a Bradford assay with bovine serum albumin as the standard (ab119216, Abcam, Melbourne, Australia), according to the manufacturer’s instructions.

### 2.3. Hepatic Metabolism

Biochemical quantification of hepatic glycogens in the liver was performed using a glycogen assay kit II (ab169558, Abcam, Melbourne, Australia). Liver tissue (10 mg) was homogenised with 200 µL of ddH_2_O for 10 min on ice. The homogenates were then boiled for 10 minutes to ensure enzyme inactivation. Following centrifugation, the supernatants were incubated with 2 µL of hydrolysis enzyme mix at room temperature for 30 min. The concentrations in the samples were interpolated from a glycogen standard curve based on optical density (OD) at 450 nm and normalised to total protein concentration.

Histological staining of lipid contents was conducted using an Oil Red O Stain Kit (ab150678, Abcam, Melbourne, Australia). Frozen liver sections (10 µm) were immersed in cold propylene glycol for 5 min and subsequently incubated with Oil Red O solution for 10 min. Slides were immersed in propylene glycol (85%) for 60 s, followed by rinsing twice with distilled water, staining with hematoxylin for 2 min, and 3 thorough rinses (once in tap water and twice in distilled water). PermaFluor^TM^ Aqueous Mounting Medium (Thermo Fisher Scientific, Fremont, CA, USA) was used to mount a cover slip. Images were captured using an Olympus VS120 slide scanner and analysed using QuPath [19].

### 2.4. Oxidative Stress and Inflammation

Carbonylated protein is commonly used to quantify protein oxidative damage and is a downstream marker of cell damage induced by reactive oxygen species from different sources [20]. Protein carbonyl content was quantified in tissue lysates based on a reaction with 2,4-dinitrophenylhydrazine derivatization using a protein carbonyl kit (ab238536, Abcam, Melbourne, Australia) and following the manufacturer’s specifications.

Myeloperoxidase (MPO) activity was measured using a MPO activity assay kit (ab111749, Abcam, Melbourne, Australia) in alignment with the manufacturer’s instructions. Snap-frozen liver samples (10 mg) were homogenised in 100 µL of cold MPO buffer. Centrifuged supernatants (15,000× *g* for 15 min at 4 °C) were collected and incubated using MPO reaction mix. The fluorescence signal was read at Ex/Em = 484/525 nm. MPO activity (fluorescence) was normalised based on protein levels (per mg).

### 2.5. DNA Damage

DNA damage was measured as the formation of apurinic/apyrimidinic (AP) sites in liver genomic DNA. Oxidisation of bases due to oxidative stress leads to a DNA repair process and AP site formation [21]. This is regarded as a key measure of DNA injury linked to oxidative stress. Genomic DNA in liver samples was isolated using a Genomic DNA Isolation Kit (ab65358, Abcam, Melbourne, Australia), while a DNA damage Assay Kit (ab211154, Abcam, Melbourne, Australia) was used to detect AP sites.

### 2.6. RNA Isolation and qPCR

Total RNA was isolated from mouse livers using a RNeasy Mini Kit (Qiagen Pty Ltd., Doncaster, Australia). Purified RNA was converted into cDNA using a QuantiTect Reverse Transcription Kit (Qiagen). Real-time amplification was performed using the QuantStudio3 Real-Time PCR System (Thermo Fisher Scientific, Scoresby, Australia). The primers for inflammatory cytokines *IL-6*, *IL-1β*, and *TNF-α* were sourced from Sigma, while the primers for three isoforms of transforming growth factor-β (*TGF-β*) (*β1*, *β2*, and *β3*) have been described previously [12], and were prepared by Integrated DNA Technologies (Singapore). Gene expression levels were normalized to the internal control, *β-actin* [12].

### 2.7. Statistical Analysis

SigmaPlot (version 13, Systat Software Inc., San Jose, CA, USA) was used for data analysis. A two-way ANOVA (treatment × sex) was used to assess differences among multiple groups. If there was no significant interaction term identified in the ANOVA, post-hoc tests were performed for the main effects (least significant differences). Differences were accepted as significant if *p* < 0.05. Data are expressed as the mean (SD) or median (range).

## 3. Results

### 3.1. Liver Metabolism

**Figure 1:**
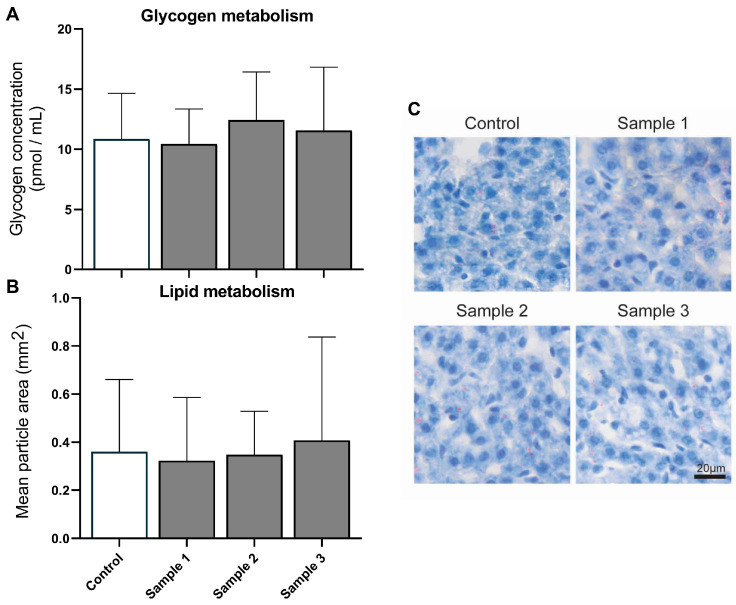
Hepatic metabolism. Glycogen (**A**) and lipid droplets (**B**) in hepatic cells were quantified and compared for the four experimental groups (saline, *n* = 26; sample 1, *n* = 20; sample 2, *n* = 19; sample 3, *n* = 20 for glycogen and saline, *n* = 9; sample 1, *n* = 13; sample 2, *n* = 15; sample 3, *n* = 14 for lipid staining). Representative lipid staining images are also shown (**C**). Values are means (SD). Scale bar = 20 µm.

To evaluate the effect of PM exposure on hepatic metabolism, we examined glucose and lipid homeostasis in mice exposed to the three residential roof space PMs (Figure 1). Compared with the controls, gestational exposure to PM did not change hepatic glycogen storage (*p* = 0.494) or lipid level (*p* = 0.893), and there was no effect of sex (*p* = 0.812 for glycogen and *p* = 0.565 for lipid).

### 3.2. Oxidative Stress and Inflammation

**Figure 2:**
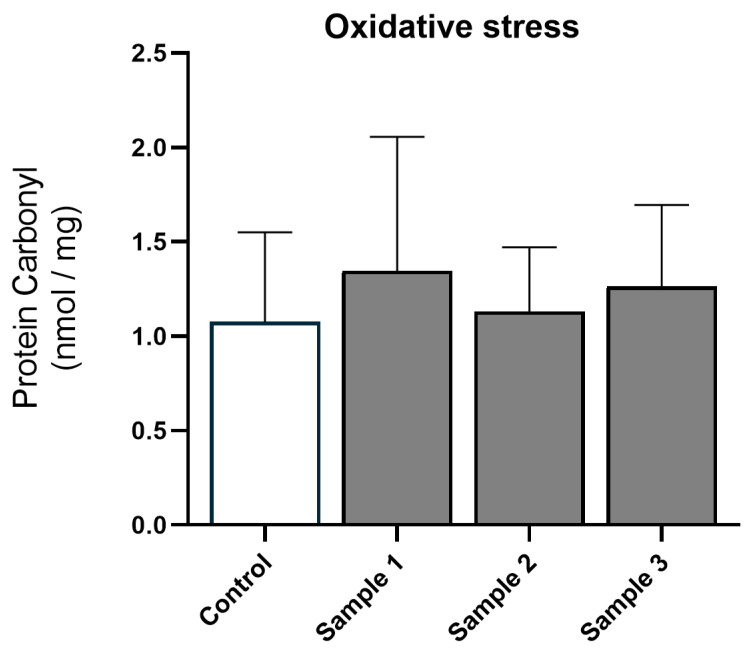
Oxidative stress: protein carbonyl levels for the four experimental groups (saline, *n* = 22; sample 1, *n* = 17; sample 2, *n* = 22; and sample 3, *n* = 22). Values are means (SD).

**Figure 3:**
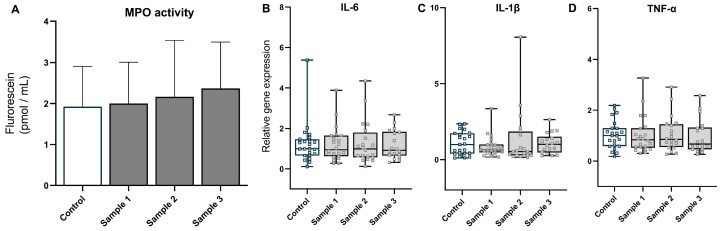
Inflammatory response: myeloperoxidase (MPO) activity (**A**) and expression of inflammatory genes (**B**–**D**) for the four experimental groups (saline, *n* = 22–23; sample 1, *n* = 19–22; sample 2, *n* = 19–20; and sample 3, *n* = 18–21). The values are the means (SD) and medians (from minimum to maximum) for MPO activity and cytokine gene expression, respectively.

After PM exposure, protein carbonyl levels (treatment *p* = 0.300, treatment × sex *p* = 0.584; Figure 2) and MPO activity (treatment *p* = 0.432, treatment × sex *p* = 0.318; Figure 3A) remained unchanged, indicating that post-natal hepatic oxidative stress and inflammation were not induced by in utero exposure to PM. Furthermore, mRNA analysis of pro-inflammatory cytokines *IL-6* (treatment *p* = 0.992, treatment × sex *p* = 0.255), *IL-1β* (treatment *p* = 0.695, treatment × sex *p* = 0.543), and *TNF-α* (treatment *p* = 0.952, treatment × sex *p* = 0.394) showed that in utero exposure to PM did not alter the expression of these genes (Figure 3B–D), confirming minimal effect of maternal PM exposure on the inflammatory response in the offspring.

### 3.3. Fibrosis

**Figure 4:**
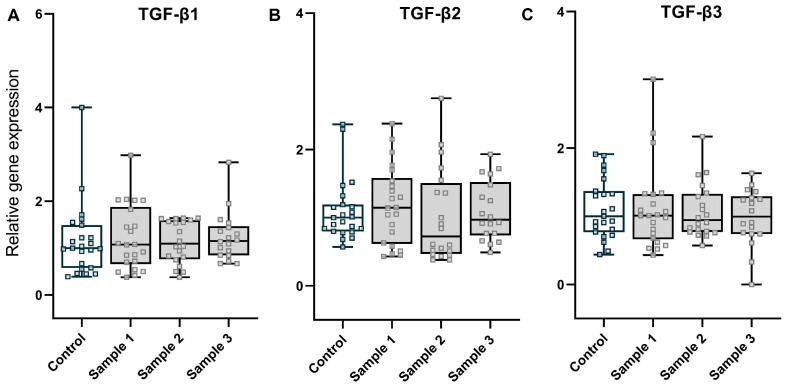
Fibrosis: expression of the central mediator genes of fibrogenesis, *TGF-β1* (**A**), *TGF-β2* (**B**), and *TGF-β3* (**C**), for the four experimental groups (saline, *n* = 23; sample 1, *n* = 22; sample 2, *n* = 20; and sample 3, *n* = 18). Values are medians (whisker indicate minimum to maximum values).

To assess the effects of PM exposure on liver fibrosis, we analysed expression of transforming growth factor β (*TGF*-*β*), which is strongly linked to fibrogenesis [12]. Similar to the other genes, we did not observe a significant difference in *TGF-β1* (treatment *p* = 0.928, treatment × sex *p* = 0.713), *TGF-β2* (treatment *p* = 0.889, treatment × sex *p* = 0.832), or *TGF-β3* (treatment *p* = 0.878, treatment × sex *p* = 0.316) mRNA expression amongst the experimental groups (Figure 4A–C). Together, these results suggest that TGF-β, an inflammatory trigger of hepatic fibrosis, was not impacted by maternal exposure to PM.

### 3.4. Genotoxicity

**Figure 5:**
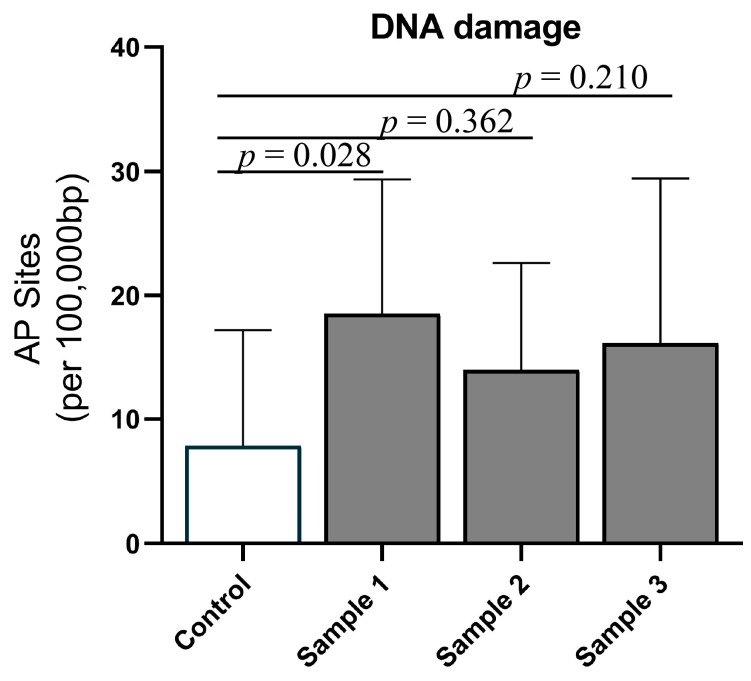
Genotoxicity: formation of apurinic/apyrimidinic (AP) sites (DNA damage) for the four experimental groups (saline, *n* = 17; sample 1, *n* = 15; sample 2, *n* = 18; and sample 3, *n* = 11). Values are means (SD). Overall, *p* = 0.032 for the treatment effect.

AP site formation was significantly increased in response to exposure to sample 1 (*p* = 0.032), but not samples 2 (*p* = 0.362), or 3 (*p* = 0.210) (Figure 5). This effect was not modified by sex (interaction *p* = 0.607). Therefore, these data suggest that prenatal roof space PM exposure causes liver DNA damage in the offspring; however, this is variable, depending on the PM sample.

### 3.5. Sex-Stratified Analysis

Sex-stratified analysis of all data further confirmed that there were no sex-specific effects (Supplementary Table S1).

## 4. Discussion

This study extends our prior observations on the link between in utero exposure to residential PM and adverse developmental outcomes in the lungs, immune system, and brain. In contrast to our prior studies [4,5], we did not observe pronounced effects of environmentally relevant PM on the developing liver by examining the hepatic metabolism and cellular response. While we found some evidence of liver genomic DNA damage in response to PM exposure, this varied depending on the PM sample and was the only indicator of an adverse response. The biological consequences of genotoxicity and its long-term effects are not clear in the context of PM exposure and are potentially worthy of further investigation.

Exposure to ambient PM with an aerodynamic diameter <2.5 µm (PM_2.5_) has been shown to trigger a non-alcoholic steatohepatitis (NASH)-like phenotype and can impair hepatic glucose and lipid metabolism in mice. This can lead to reductions in glycogen storage and increases in hepatic triglycerides and cholesterol [11]. Unlike these direct exposure effects, which are well-established, we did not observe any significant changes in hepatic glycogen or lipids after in utero exposure to community sampled PM. Previous studies using different models have indicated that PM exposure might act as a risk factor for the progression of liver injury (e.g., NAFLD). For example, epidemiological studies provide evidence that long-term exposure to ambient PM might interact with unhealthy lifestyle habits (e.g., a high-fat diet) and central obesity, contributing to a high prevalence of metabolic dysfunction-associated fatty liver disease (MAFLD) [7]. Similarly, in vivo animal work has suggested that the effect of a high-fat diet on metabolic syndrome is enhanced by concomitant exposure to PM_2.5_ [22,23], while in vitro studies show that PM_2.5_ exposure activates Toll-like receptor (TLR) 4 in Kupffer cells to enhance inflammatory potential and increase hepatic stellate cell collagen synthesis and fibrosis [24].

Inflammatory and oxidative stress responses are strongly associated with organ injury induced by inhalation of PM_2.5_. Indeed, prolonged (24 weeks) PM_2.5_ exposure in mice has been shown to induce hepatic inflammation and oxidative stress, contributing to abnormal hepatic function and lipid accumulation [25]. Interestingly, this effect was not observed early in the exposure protocol (3 weeks), and an extended exposure period (10 weeks) was necessary in order for the inflammatory cascade to be triggered via JNK-AP1, NF-ĸB, and TLR4 [11]. Thus, exposure duration seems to be critical in determining the adverse cellular response. In the current study, the pregnant mice were exposed to PM from mid- to late gestation, which is a known period of sensitivity to the effects of air pollution [4,5,15,16,17,18]. Our data showed that the level of carbonylated proteins (a marker of oxidative stress), myeloperoxidase (MPO) activity (a parameter for tissue inflammation), and inflammatory cytokine expression (*IL-6*, *IL-1β*, and *TNF-α*) remained unchanged, suggesting minimal impact of maternal PM exposure on inflammaion and oxidative stress in post-natal liver development.

Hepatic fibrosis is a key feature of chronic liver injury. PM_2.5_ exposure itself is sufficient to cause the early stages of hepatic fibrosis in murine models, with these effects being exacerbated by exposure to a high-fat diet [12]. The underlying mechanism has been attributed to upregulation of hepatic transforming growth factor β expression, leading to activation of SMAD3 signalling and increased collagen production [12]. However, our results did not support evidence of fibrogenic dysregulation, as evidenced by the fact that *TGF-β* mRNA levels remained similar after in utero exposure to PM.

Many PM components have been classified as carcinogens. Despite a lack of epidemiological evidence linking maternal air pollution to the post-natal development of liver cancers, organisms undergoing rapid development are thought to display increased sensitivity to PM toxicity. During development, while the immune system is immature, there is only a short time for DNA repair to occur during cell division. As a result, early-life exposure to environmental insults can imprint cell phenotypes, leading to an increased risk of chronic disease later in life, such as cancer [26]. One study has shown that carbon nanoparticles can induce DNA strand breaks in offspring exposed in utero [6]. We had some evidence to support this with the upregulation of markers of DNA damage in response to one of our samples. This effect was consistent between sexes but PM sample-dependent, which may not be surprising given the complex and heterogeneous components of the roof space PM samples collected. Thus, it is likely that the sample-specific genotoxicity effect is due to the chemical composition of the samples. However, PM size and size distribution are also critical in determining the detrimental cellular response. The lack of PM size measurements is a major limitation of the current study and requires further investigation. Certainly, follow-up investigations are also needed to evaluate long-term DNA damage and repair and determine the genotoxic consequences. In addition, oxygen-derived free radicals are usually considered to be the primary drivers of genotoxicity [27]. As described earlier, we did not find noticeable oxidative stress by quantifying oxidated protein as surrogate for oxidative stress. Since there is variability in the capacity of different reactive oxygen species (ROS) to induce oxidative DNA damage, lipid peroxidation, and protein modification [20], it is of great importance to ascertain and directly measure the predominant ROS to understand the mechanism(s) that could mediate genotoxicity.

## 5. Conclusions

In summary, we showed that maternal exposure to residential PM during mid-late gestation, when used as a surrogate for ambient PM sources, did not interrupt post-natal glycogen and lipid metabolism or induce tissue inflammation, protein oxidation, or fibrosis in the developing liver. However, we did find some evidence of a genotoxic effect. Taken together, these data indicate that maternal exposure to residential PM may only have mild effects on post-natal liver development, particularly compared with other organs/systems, such as the lung, brain, and immune system, where we have observed significant detrimental effects using the identical protocol [4,5]. Given that different exposure regimes may impact the outcome, such as variations in dose, exposure timing, and duration of exposure, further study in this emerging field is warranted.

## Data Availability

The data presented in this study are available on request from the corresponding author.

## Supplementary Materials

The following supporting information can be downloaded at: https://www.mdpi.com/article/10.3390/toxics11010061/s1, Table S1: Sex-stratified analysis of all data.
