# Supplementary material for: Can Maternal Exposure to Air Pollution Affect Post-Natal Liver Development?
Source: Toxics. 2023 Jan 9;11(1):61. doi: 10.3390/toxics11010061 (PMC9866810; doi:10.3390/toxics11010061)
Supplement: Supplementary file 1 [file toxics-11-00061-s001.zip › toxics-2117129-supplementary.pdf]

Supplementary Table S1: Sex-stratified analysis of all data.

		Female			Male		
	**Group**	**n**	**Value**	**Overall *p***	**n**	**Value**	**Overall *p***
**Weight** (mg)	Control	13	6.47 (1.12)	0.422	12	6.92 (0.74)	0.330
	Sample 1	13	5.95 (0.71)		12	6.43 (0.88)	
	Sample 2	12	6.27 (0.70)		10	6.46 (0.69)	
	Sample 3	12	6.28 (0.43)		10	6.67 (0.38)	
**SVL** (mm)	Control	13	49.42 (2.96)	0.449	12	50.97 (2.25)	0.150
	Sample 1	13	48.08 (2.59)		12	49.01 (2.44)	
	Sample 2	12	49.51 (1.85)		10	49.48 (2.45)	
	Sample 3	12	48.93 (2.25)		10	49.34 (1.35)	
**Glycogen** (pmol / mL)	Control	14	10.27 (3.71)	0.460	12	11.48 (3.98)	0.808
	Sample 1	10	10.69 (2.29)		10	10.19 (3.52)	
	Sample 2	11	12.89 (3.27)		8	11.81 (4.99)	
	Sample 3	12	11.47 (6.24)		8	11.70 (3.70)	
**Lipid** (mm^2)	Control	2	0.56 (0.11)	0.779	7	0.30 (0.32)	0.569
	Sample 1	7	0.40 (0.34)		6	0.23 (0.11)	
	Sample 2	10	0.31 (0.14)		5	0.43 (0.24)	
	Sample 3	9	0.43 (0.52)		3	0.33 (0.23)	
**Protein carbonyl** (nmol / mg)	Control	11	1.16 (0.50)	0.847	11	0.99 (0.45)	0.209
	Sample 1	9	1.24 (0.69)		8	1.47 (0.77)	
	Sample 2	12	1.18 (0.39)		10	1.07 (0.28)	
	Sample 3	12	1.33 (0.44)		10	1.18 (0.43)	
**MPO activity** (pmol / mL)	Control	12	1.91 (1.07)	0.806	10	2.02 (0.93)	0.264
	Sample 1	10	2.06 (0.96)		9	1.94 (1.11)	
	Sample 2	10	2.43 (1.80)		9	1.87 (0.68)	
	Sample 3	12	2.10 (1.06)		9	2.71 (1.19)	
IL-6 (Relative gene expression)	Control	12	0.99 (0.51)	0.593	11	1.53 (1.36)	0.548
	Sample 1	10	1.53 (1.07)		12	0.96 (0.55)	
	Sample 2	11	1.30 (1.20)		9	1.26 (0.95)	
	Sample 3	10	1.20 (0.80)		8	1.18 (0.62)	
IL-1β (Relative gene expression)	Control	12	0.83 (0.62)	0.743	11	1.32 (0.83)	0.279
	Sample 1	10	1.04 (0.94)		12	0.71 (0.36)	
	Sample 2	11	1.45 (2.36)		9	1.12 (1.13)	
	Sample 3	10	1.00 (0.76)		8	1.16 (0.57)	
TNF-α (Relative gene expression)	Control	12	0.91 (0.50)	0.508	11	1.20 (0.60)	0.755
	Sample 1	10	0.69 (0.33)		12	1.29 (0.86)	
	Sample 2	11	0.98 (0.61)		9	1.17 (0.81)	
	Sample 3	10	1.03 (0.61)		8	0.92 (0.77)	
TGF-β1	Control	12	1.19 (1.01)	0.972	11	1.14 (0.47)	0.620

(Relative gene expression)	Sample 1	10	1.20 (0.77)		12	1.24 (0.65)	
	Sample 2	11	1.13 (0.45)		9	1.15 (0.48)	
	Sample 3	10	1.06 (0.30)		8	1.46 (0.68)	
TGF-β2 (Relative gene expression)	Control	12	1.16 (0.59)	0.691	11	1.03 (0.25)	0.930
	Sample 1	10	1.19 (0.58)		12	1.18 (0.59)	
	Sample 2	11	0.92 (0.72)		9	1.14 (0.66)	
	Sample 3	10	1.07 (0.31)		8	1.14 (0.58)	
TGF-β3 (Relative gene expression)	Control	12	1.10 (0.47)	0.474	11	1.11 (0.42)	0.565
	Sample 1	10	0.83 (0.26)		12	1.35 (0.74)	
	Sample 2	11	1.04 (0.36)		9	1.12 (0.46)	
	Sample 3	10	0.94 (0.52)		8	1.03 (0.26)	
**DNA damage** (AP sites)	Control	9	7.42 (9.35)	0.097	8	8.34 (9.97)	0.338
	Sample 1	7	22.69 (11.20)		8	14.91 (9.66)	
	Sample 2	10	16.14 (10.45)		8	11.24 (5.09)	
	Sample 3	6	15.87 (16.60)		5	16.40 (9.97)	

Values are Mean (SD). SVL: snout-vent length, MPO: myeloperoxidase.
